# Sphingosine-1-Phosphate Induces the Migration of Thyroid Follicular Carcinoma Cells through the MicroRNA-17/PTK6/ERK1/2 Pathway

**DOI:** 10.1371/journal.pone.0119148

**Published:** 2015-03-06

**Authors:** Shitao Zhao, Jincheng Li

**Affiliations:** The Department of Breast and Thyroid Sugery, The First Affiliate Hospital of Liaoning Medical University. Jinzhou, Liaoning, China; UCSF / VA Medical Center, UNITED STATES

## Abstract

Sphingosine-1-phosphate (S1P) is a bioactive lipid known to play a role in tumorigenesis and cancer progression. However, the molecular mechanisms of S1P regulated migration of papillary thyroid cancer cells are still unknown. In this study, we showed that S1P induced PTK6 mRNA and protein expression in two thyroid follicular cancer cell lines (ML-1 and FTC-133). Further studies demonstrated that induced PTK6 and its downstream signal component (ERK1/2) are involved in S1P-induced migration. Upon investigating the mechanisms behind this event, we found that miR-17 inhibited the expression of PTK6 through direct binding to its 3’-UTR. Through overexpression and knockdown studies, we found that miR-17 can significantly inhibit S1P-induced migration in thyroid follicular cancer cells. Interestingly, overexpression or knockdown of PTK6 or ERK1/2 effectively removed the inhibition of S1P-induced migration by miR-17. Furthermore, we showed that S1P decreased miR-17 expression levels. Meanwhile, in papillary thyroid cancers, miR-17 is downregulated and negatively associated with clinical staging, whereas PTK6 is upregulated and positively associated with clinical stages. Collectively, our work defines a novel signaling pathway implicated in the control of thyroid cancer migration.

## Introduction

Sphingosine-1-phosphate (S1P) is a bioactive sphingolipid metabolite that is produced by various cell types, including platelets, glioma cells and fibroblasts [1–3]. The secreted S1P binds to a family of five G-protein coupled receptors, S1P_1−5,_ resulting in endothelial proliferation, migration and angiogenesis [4, 5]. A recent report demonstrated that S1P regulated the migration of human thyroid cancer cells, implying that S1P plays an important role in thyroid tumour growth and metastasis [6].

Protein tyrosine kinase 6 (PTK6), also known as breast tumor related kinase (BRK), is an intracellular tyrosine kinase highly expressed in human breast tumors [7, 8]. It is distantly related to the c-Src kinase family, possessing an SH3 domain, an SH2 domain and a catalytic tyrosine kinase domain [9–11]. PTK6 has been identified as the most common aberration in human invasive ductal breast tumours, as it is detectable in over 80% of cases [8, 12]. The molecular mechanisms of PTK6 in promoting growth factor signaling, proliferation, and migration have been discovered through the identification of many PTK6 substrates and interacting proteins [13–16]. However, a clear role for PTK6 in cancer has not been well-studied.

MicroRNAs (miRNAs) are endogenous, noncoding, small RNAs (∼22 nucleotides in length) that are involved in posttranscriptional control of gene expression [17–19]. The polycistronic microRNA cluster miR-17∼92 encodes six members (miR-17, miR-18a, miR-19a, miR-20a, miR-19b-1 and miR-92-1) [20]. Recently, studies have revealed that miR-17∼92 may be involved in heart development, apoptosis and haematopoietic malignancies. For example, loss of miR-17∼92 results in upregulation of Bim and increased apoptosis, inhibiting the pro-B to pre-B transition [21]. Volinia et al showed that miR-17∼92 was associated with haematopoietic malignancies [22]. Although several functions of miR-17∼92 have been described, a clear role for miR-17 in thyroid follicular carcinoma has not been established.

In this study, we sought to determine the role of S1P in PTK6, miR-17 and ERK1/2 expression and to determine whether S1P regulates the migration of papillary thyroid cancer cells via a miR-17 /PTK6/ ERK1/2 signal.

## Materials and Methods

### Ethics statement

All participants gave written informed consent to participate in the study. The study was conducted according to the principles of the Declaration of Helsinki and approved by the Institutional Review Board of the first affiliate hospital of liaoning medical university, in accordance with its guidelines for the protection of human subjects.

### Samples and cases

PTC samples and adjacent nontumorous tissues (located>3cm away from the tumor) were collected from 162 patients who undergoing surgery at the first affiliate hospital of liaoning medical university from February 2010 to February 2014. Tissue samples were cut into two parts, one was reviewed by two expert pathologists to verify the histologic diagnosis, the other immediately snap-frozen in liquid nitrogen, and stored in liquid nitrogen until RNA extraction. None of the patients had received any preoperative treatment. Tumors were staged according to the American Joint Committee on Cancer (AJCC) pathologic tumor-node-metastasis (TNM) classiication. The characteristics of patients are described in S1 Table.

### Reagents and cell culture

ML-1 thyroid follicular cancer cells grown in DMEM with 2mM L-glutamine, 10% (v/v) FCS and 100 units/ml of penicillin and streptomycin at 37°C with 5% carbon dioxide. FTC-133 human follicular thyroid cancer cells were grown in Ham’s medium and DMEM (1:1) supplemented with 10% FBS, 2 mM L-glutamine and 100 U/ml penicillin/streptomycin. All cells were provided by the China Center Type Culture Collection and incubated at 37°C with 5% carbon dioxide.

U0126, S1P, antibody against PTK6, β-actin, ERK1/2, p-ERK1/2 were purchased from Sigma (St. Louis, MO, USA). Foetal calf serum was purchased from GIBCO (Life Technologies Europe, Switzerland). Transwell inserts was purchased from Corning (NY, USA)). MiR-17 precursor (Pre-miR-17), miRNA precursor control (pre-control), antisense miR-17 oligonucleotide (anti-miR-17), antisense miRNA control (anti-control), were purchased from Ambion (Austin, TX, USA).

### Cell migration

These experiments were performed as described [6, 23].

### Transfection and luciferase reporter gene assays

Cells were seeded on 24-well dishes and transfected by using Lipofectamine 2000 (Invitrogen) for 24 hours, after which they were serum-starved for an additional 24 hours prior to harvest. Transfection complexes were prepared according to the manufacturer’svinstructions A Renilla luciferase reporter vector pRL-TK was used as internal control. Luciferase assays were performed with a dual-specific luciferse assay kit (Promega). Firefly luciferase activities were normalized on the basis of Renilla luciferase activities.

### Quantitative RT-PCR Analysis of PTK6 and ERK mRNA Expression

Total RNA was isolated using TRIzol (Invitrogen, Basel, Switzerland). Cellular RNA samples were reverse-transcribed using random primers. Quantitative RT-PCR (real-time PCR) was performed using an Icycler Instrument (Bio-rad, CA, USA) and iQ-SYBR Green Supermix (Bio-rad, CA, USA). GAPDH was amplified as an internal control. Primers used this study are listed in S2 Table.

### Detection of Mature MicroRNAs by TaqMan Real-time RT-PCR

Real-time RT-PCR analysis for mature miR-17, 509–1, 509–2, 509–3, 452, 622, 93, 106b, 26a-1, 26a-2, 93b, 520g, 520h, 372, 331, 650, 20b were respectively carried out in triplicate using TaqMan MicroRNA assays kit (Ambion) according to manufacturer’s instruction. U6 snRNA was used as the endogenous control.

#### Western Blot Analysis

Whole-cell lysates were prepared by lysing cells with radioimmunoprecipitation assay (RIPA) buffer (100 mMTris, pH 7.4, 150 mM NaCl, 5 mM EDTA, 1% Triton X-100, 1% deoxycholate acid, 0.1% SDS) supplemented with protease inhibitors cocktail (Roche). Protein concentrations were measured by BCA assay. The polypeptides from cell lysates were separated on SDS/12% polyacrylamide gels cross-linked with N,N-methylenebisacylamide, and transferred electrically to nitrocellulose membranes. Nonspecific sites were blocked with 5% nonfat dried milk before being incubated with an Antibody used in this study. Blots were developed using SuperSignal Chemiluminescent reagent (Pierce, Rockford, IL, USA), and analyzed the stained membranes with a LAS-4000 image document instrument (FujiFilm, Tokyo, Japan).

### RNA interference

PTK6 small interfering RNAs (siRNA-PTK6), ERK small interfering RNAs (siRNA-ERK) and those negative control were synthesized by RiBo Biotech (GuangZhou RiBo Biotech). Those target sequences are listed in S3 Table.

### Gene silencing with the lentivirus encoding specific shRNA

pLKO.1 vector, pLKO.1-control shRNA, pLKO.1-PTK6 shRNA plasmids were purchased from Sigma (St. Louis, MO, USA). pLKO.1-PTK6 shRNA plasmids and lentivirus packaging plasmids (pCMV-ΔA.9 and pCMV-VSVG) were cotransfected into 293 cells with Lipofectamine 2000 (Invitrogen). The virus was harvested in 3 consecutive days and filtered with filters (Millipore Corp, USA). Before infection, the same volume of fresh medium containing polybrene (8 μg/mL) were added into the virus-containing media, which were used to replace the culture media of interested cells. After 24 h, the virus-infected cells were selected by puromycin (1 μg/mL) for 48 h and subjected to required assays.

### Electrotransfection

ML-1 and FTC-133 cells were transfected with miRNA, siRNA or plasmid DNA by electroporation with an Amaxa Nucleofector II Device according to the manufacturers’ protocols.

#### Statistical Analysis

All experiments were repeated at least three times with similar results. Representative data are shown. Statistical analyses were performed using paired Student’s T-tests. A value of P < 0.05 was considered statistically significant.

## Results

### S1P induces PTK6 expression in ML-1 and FTC-133 cells

**Fig 1:**
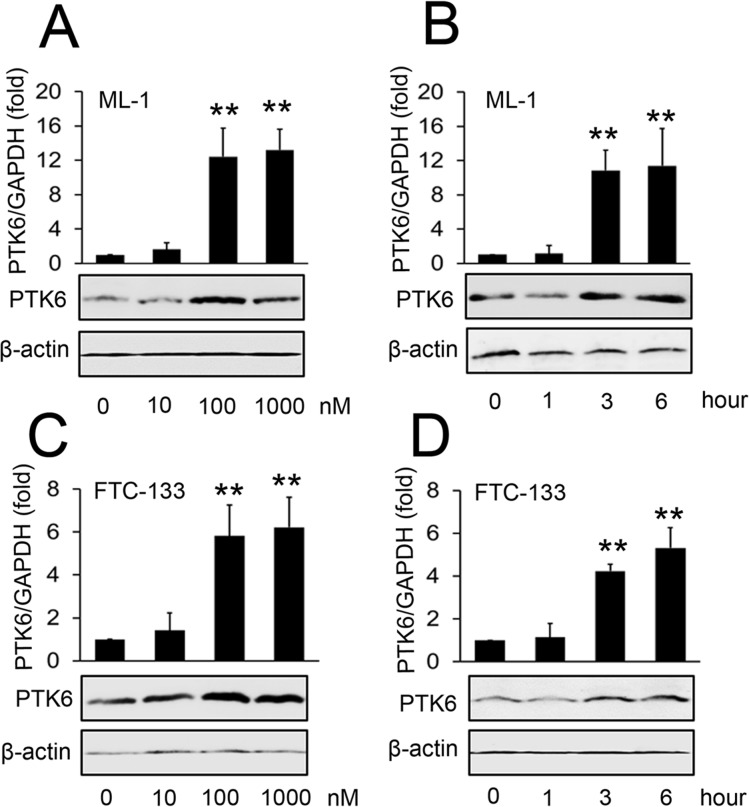
Effect of S1P on the expression of PTK6. (A, C) ML-1 cells (A) and FTC-133 cells (C) were stimulated with the indicated concentrations of S1P for 3 hours. PTK6 RNA levels were quantified by real-time RT-PCR (upper panel) and protein levels of PTK6 were detected by Western blot (lower panel). (B, D) ML-1 cells (B) and FTC-133 cells (D) were stimulated with 100 nM S1P for the indicated times. PTK6 RNA levels were quantified by real-time RT-PCR (upper panel) and protein levels of PTK6 were detected by Western blot (lower panel). Experiments were performed three times with similar results (lower panels of A-D). Data represent means±SD, n = 3 (**P < 0.01; *P < 0.05).

It has reported that treating ML-1 thyroid cancer cells with S1P significantly induces their migration [6]. Another study has shown that PTK6 promotes the migration of pancreatic cancer cells [24]. In light of these two reports, we investigated whether S1P could affect PTK6 expression. We found that S1P stimulated ML-1 cells result in increased PTK6 mRNA and protein expression. Increased levels of PTK6 mRNA were detected as early as three hours following treatment with 100 nm S1P (Fig. 1A and B, upper panel). PTK6 protein levels showed a similar trend as determined by Western blot (Fig. 1A and B, lower panel). We also showed that S1P increased PTK6 mRNA and protein expression in a dose- and time-dependent manner in follicular thyroid cancer cells (FTC-133 cells) (Fig. 1C and D). It has been reported that ERK signaling is a critical mediator in PTK6-promoted migration [24]. To investigate whether S1P could affect ERK signaling, ML-1 cells were treated with or without 100nM S1P for three hours. Western blot assays indicated that S1P induced the activation of ERK1/2 (S1 Fig.). These results show that S1P stimulates PTK6 expression and activates its downstream signaling molecules.

### PTK6 mediated S1P-induced migration

**Fig 2:**
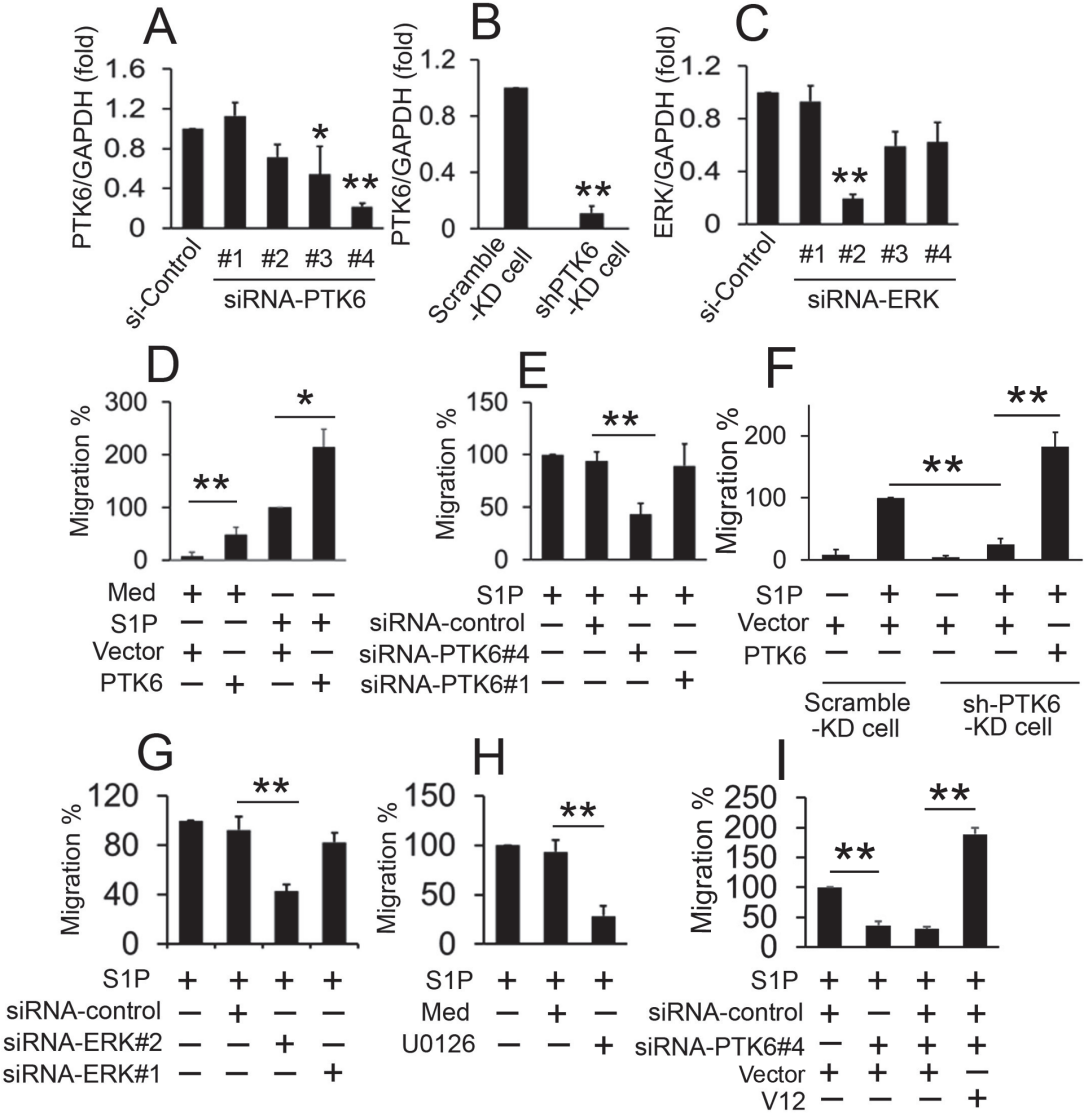
Effect of PTK6 and ERK1/2 on S1P-induced migration of ML-1 cells. (A) ML-1 cells were transfected with siRNA-control or indicated siRNA-PTK6s for 48 hours prior to real-time RT-PCR analysis. (B) Real-time RT-PCR analysis was used to determine the expression of PTK6 2 days after infection of ML-1 cells with lentiviral vector pLKO.1 carrying shRNA for PTK6 (shPTK6-KD cell) or control shRNA (Scramble-KD cell). (C) Experiments were performed as in A except cells were transfected with indicated siRNA-ERKs. (D) ML-1 cells were transfected with pcDNA3.1-Mock (empty vector) or pcDNA3.1-PTK6 for 24 hours. Then, cells were treated with or without S1P (100 nM, 30 min) and allowed to migrate towards serum for 12 hours. (E) Experiments were performed as in D except that the indicated siRNA-PTK6 were used. (F) Scramble-KD cells and shPTK6-KD cells were transfected with the indicated expression plasmids for 24 hours and treated with or without S1P (100 nM, 30 min) and allowed to migrate towards serum for 12 hours. (G) Experiments were performed as in D, except the indicated siRNA-ERK were used. (H) ML-1 cells were treated with UO126 (10 μM) or DMSO (Med) for 12 hours and treated with S1P (100 nM, 30 min) and allowed to migrate towards serum for 12 hours. (I) ML-1 cells were transfected with the indicated plasmids and siRNA for 24 hours and treated with S1P (100 nM, 30 min) and allowed to migrate towards serum for 12 hours. In the real-time RT-PCR experiments, the control was designated as 1. All experiments were repeated at least three times with similar results. Bar graphs represent means±SD, n = 3 (**P < 0.01; *P < 0.05).

To determine whether PTK6 was involved in the regulation of S1P triggered migration, we examined the effect of altering PTK6 expression on S1P-triggered migration. We designed four specific small interfering RNAs (siRNAs) for PTK6 (siRNA-PTK6#1, #2, #3 and #4) (Fig. 2A). We also knocked down PTK6 in ML-1 cells with PTK6 short hairpin RNA (shRNA) in a lentiviral system to ensure specific and stable gene silencing (Fig. 2B). In cell migration experiments, overexpression of PTK6 promoted S1P-induced migration in ML-1 cells (Fig. 2D). Conversely, PTK6 knockdown by siRNA-PTK6 #4 strongly inhibited S1P-induced migration in ML-1 cells (Fig. 2E). To determine whether PTK6-mediated S1P-induced migration is a common feature in follicular thyroid cancer cells, similar experiments were performed in FTC-133 cells (S2A and B Fig.). S1P-induced migration was also strongly attenuated in shRNA-PTK6-KD cells and the inhibitory efficiencies were abolished by transfecting PTK6 overexpression plasmids (Fig. 2F).

It has been reported that PTK6-promoted cancer migration occurs through ERK signaling [24], thus, we sought to determine whether ERK signaling also played a role in S1P-triggered migration. We constructed multiple siRNAs for ERK (siRNA-ERK#1, #2, #3 and #4) and examined the efficiency of those siRNAs (Fig. 2C). Cell migration results showed that siRNA-ERK#2 markedly inhibited S1P-triggered migration, but siRNA-ERK#1 did not affect S1P-triggered migration due to low inhibition efficiency (Fig. 2G). We next used U0126, a specific selective inhibitor of ERK1/2, to inhibit ERK1/2 activity. As shown in Fig. 2H, S1P-induced migration was reduced by U0126. This effect is not cell type specific because knockdown of ERK or treatment with U0126 also inhibited S1P-induced migration in FTC-133 cells (S2C and D Fig.). On other hand, we used Plasmid V12, encoding activated Ha-Ras [25], a mutant form of Ras possessing irreversible GTP-binding activity, to stimulate the ERK1/2 activity. As shown in Fig. 2I, siRNA-PTK6#4 inhibited S1P-triggered migration in ML-1 cells as expected, but ML-1 cells transfected with V12 completely abolished effects of siRNA-PTK6#4. Similar results were also obtained in FTC-133 cells (S2E Fig.). All of these results demonstrate that PTK6 and ERK1/2 signaling are involved in the migration of follicular thyroid cancer cells in response to S1P stimulation.

### Identification of miRNAs targeting the 3’-UTR of PTK6

To identify miRNAs that control the expression of luciferase through targeting the 3’-UTR of PTK6, we constructed a luciferase reporter plasmid that contains the 3’-UTR of PTK6. Using two widely utilized programs, miR Walk and Target Scan, we predicted that 17 miRNAs could target the 3’-UTR of PTK6. We next investigated whether those miRNAs played a role in PTK6 expression. In reporter assays, seven miRNAs reduced luciferase activity below 70% of that in transfected control cells, suggesting that these miRNAs negatively regulate gene expression via the 3’-UTR of PTK6 (S3A Fig.). To explore which miRNAs were involved in S1P-induced signaling, ML-1 cells were treated with S1P at 100 nm for three hours. Real-time RT-PCR assays indicated that, among those miRNAs, only the expression of miR-17 was significantly changed following S1P treatment (S3B Fig.). These results indicate that miR-17 could play a role in the S1P/PTK6 signal pathway.

### PTK6 is a direct target gene of miR-17

**Fig 3:**
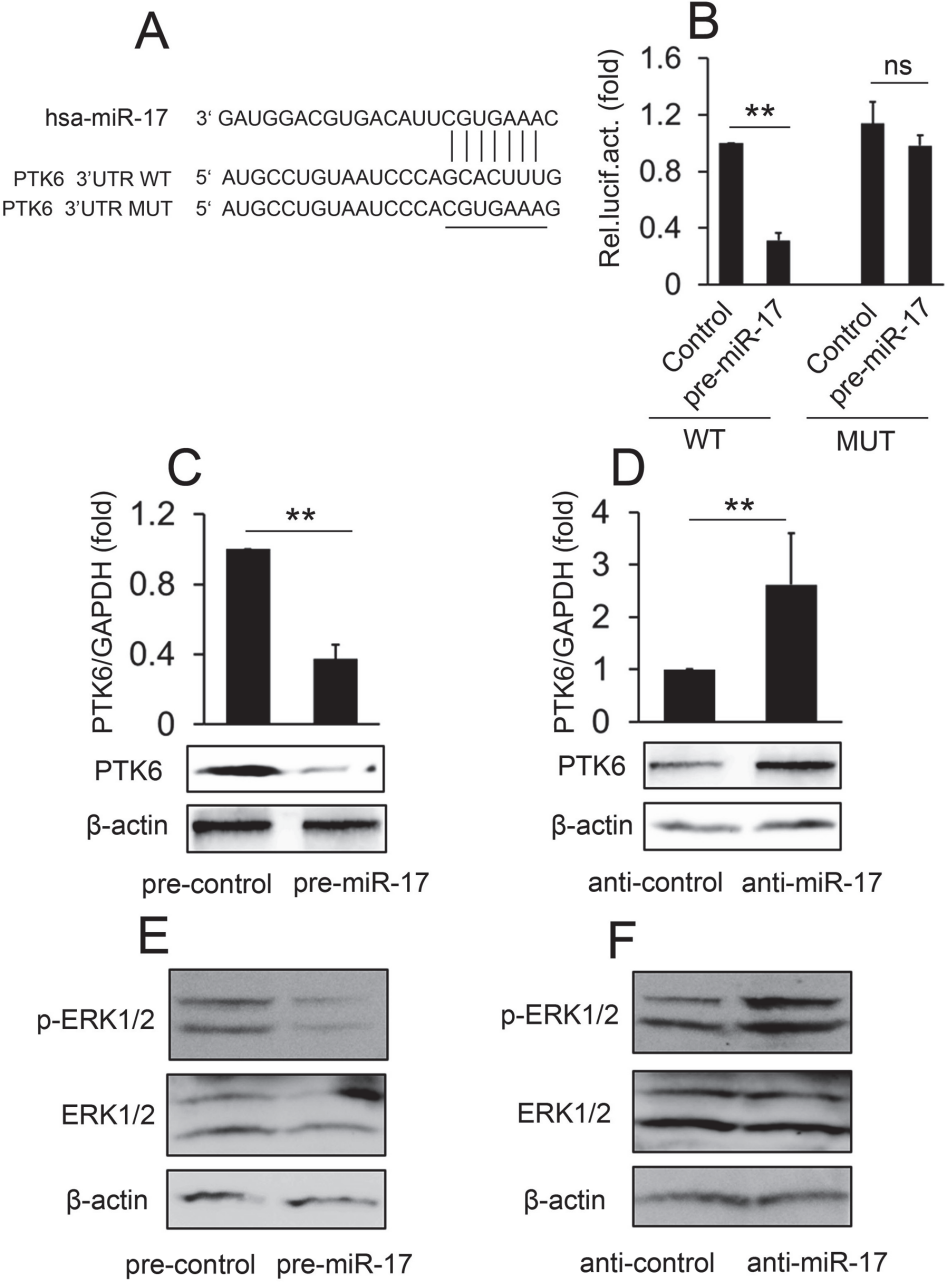
miR-17 down-regulates PTK6 expression by directly targeting its 3’UTR. (A) The sequences of miR-17 binding sites within the human PTK6 3’UTRs and schematic reporter constructs, in this panel, PTK6-WT represents the reporter constructs containing the entire 3’UTR sequences of PTK6. PTK6-MUT represent the reporter constructs containing mutated nucleotides. (B) 293T cells were co-transfected with WT or MUT PTK6 3’UTR reporter plasmids and pre-miR-17 for 48 hours prior to luciferase assays. (C) ML-1 cells were transfected with pre-control or pre-miR-17 for 48 hours prior to real-time RT-PCR assays (upper panel) and Western blot assays (lower panel). (D) Experiments were performed as in C, except anti-control and anti-miR-17 were used. (E) ML-1 cells were transfected with pre-control or pre-miR-17 for 48 hours prior to western blot assays. (F) Experiments were performed as in E except anti-control and anti-miR-17 were used. All experiments were repeated at least three times with similar results. Bar graphs represent means±SD, n = 3 (**P < 0.01; *P < 0.05).

Since miR-17 was likely involved in the S1P/PTK6 signal pathway, we sought to elucidate the molecular mechanisms by which miR-17 executes its function. We searched for potential targets of miR-17 in the 3’-UTR of PTK6 using different computational methods, and then constructed wild-type PTK6 3’-UTR and mutated reporter plasmids (Fig. 3A). Reporter assays indicated that co-transfection of pre-miR-17 and the wild-type PTK6 3’-UTR caused a significant decrease in luciferase activity relative to the controls (Fig. 3B). However, the co-transfection of miR-17 and the mutant PTK6 3’-UTR did not affect luciferase activity (Fig. 3B). These results suggest that miR-17 directly targets the PTK6 3’-UTR.

We also investigated the effect of miR-17 on endogenous PTK6 expression. Compared to cells transfected with pre-control, pre-miR-17-transfected ML-1 cells showed reduced PTK6 expression evidenced by both real-time RT-PCR and Western blot experiments (Fig. 3C). To further validate the effect of miR-17 on PTK6 in ML-1 cells, we neutralized endogenously expressed miR-17 using an antisense oligonucleotide (anti-miR-17). Following miR-17 silencing, PTK6 mRNA and protein levels were upregulated (Fig. 3D). The role of miR-17 on PTK6 expression was confirmed by repeating the experiments using FTC-133 cells (S4A and B Fig.). We next investigated the role of miR-17 in the regulation of PTK6 expression mediated by S1P. The expression of PTK6 was also increased in FTC-133 cells after treatment with S1P, and this elevation in PTK6 was reversed by miR-17, at both the mRNA and protein levels (S4C Fig.). We further examined whether ERK1/2 activity was affected by miR-17. Western blot analysis indicated that overexpression of miR-17 inhibited ERK1/2 activity and that inhibition of miR-17 enhanced ERK1/2 activity (Fig. 3E and F). Taken together, these results suggest that miR-17 down-regulates PTK6 expression and ERK1/2 activity by directly targeting PTK6 3’ UTR.

### The role of miR-17 targeting of PTK6 in the S1P-induced migration of follicular thyroid cancer cells

**Fig 4:**
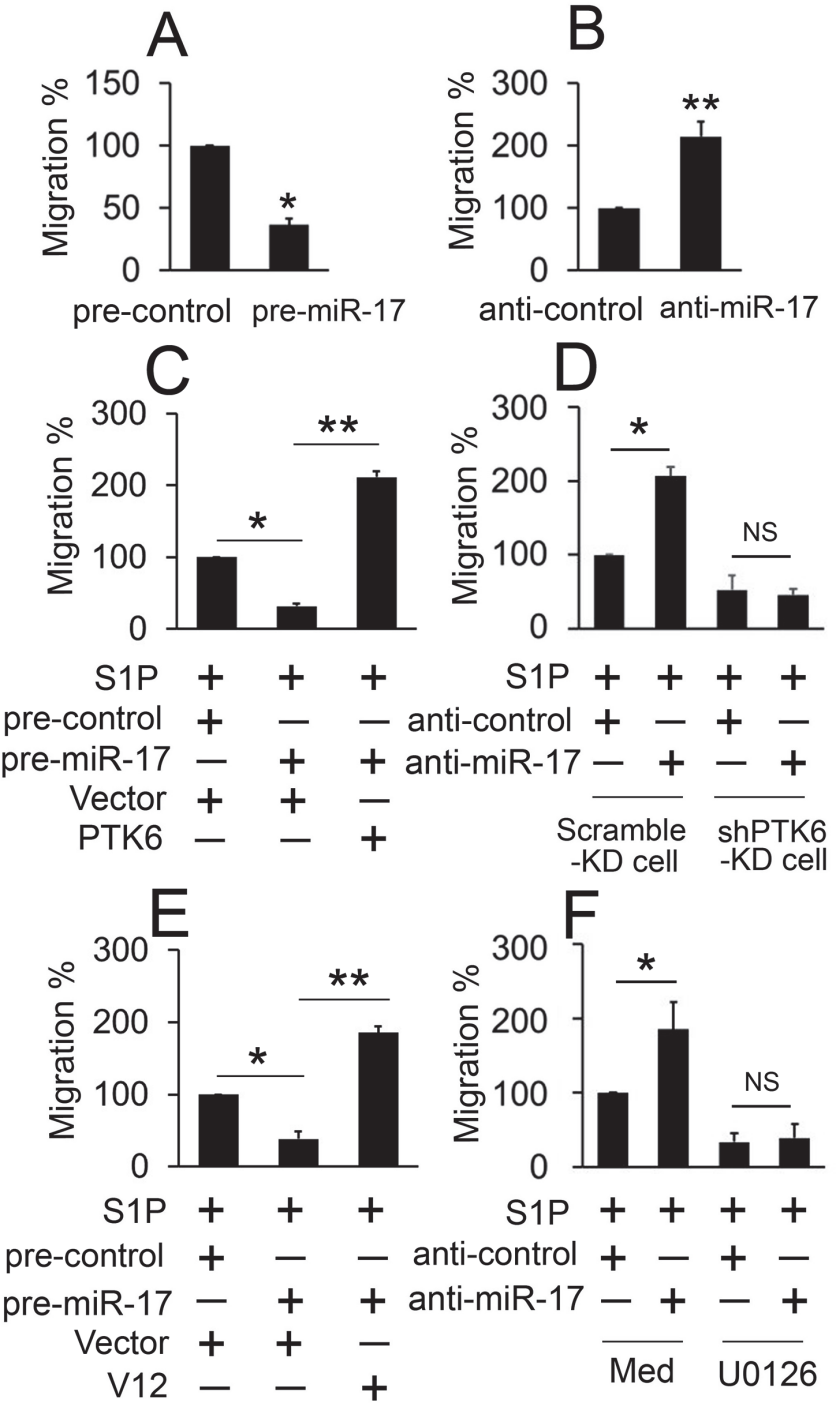
miR-17 inhibits the S1P-induced cell migration through the PTK6-ERK signal pathway. (A) ML-1 cells were transfected with pre-miR-17 or pre-control for 24 hours and treated with S1P (100 nM, 30 min) and allowed to migrate towards serum for 12 hours. (B) Experiments were performed as in A except anti-control and anti-miR-17 were used. (C) ML-1 cells were transfected with the indicated plasmids and miRNAs for 24 hours and treated with S1P (100 nM, 30 min) and allowed to migrate towards serum for 12 hours. (D) Experiments were performed as in B except scramble-KD cells and shPTK6-KD cells were used. (E) Experiments were performed similarly as in C except V12 were used. (F) ML-1 cells were transfected with anti-miR-17 or anti-control for 24 hours, then treated with UO126 (10 μM) or DMSO (Med) for 12 hours and treated with S1P (100 nM, 30 min) and allowed to migrate towards serum for 12 hours. All experiments were repeated at least three times with similar results. Bar graphs represent means±SD, n = 3 (**P < 0.01; *P < 0.05).

To examine the function of miR-17 in follicular thyroid cancer cell migration, ML-1 cells were transfected with pre-miR-17 and a scrambled control sequence. As shown in Fig. 4A, cells transfected with pre-miR-17 were distinctively less migratory than control cells after S1P induction. Conversely, miR-17 inhibitor significantly increased S1P-induced ML-1 cell migration (Fig. 4B). Furthermore, pre-miR-17 also decreased S1P-induced migration and miR-17 inhibitor enhanced S1P-induced migration in FTC-133 cells (S5A and B Fig.).

We next examined the functional relevance of the miR-17/PTK6 interaction in follicular thyroid cancer cells. Results from the cell migration experiments confirmed that overexpression of PTK6 impaired miR-17-reduced migration in ML-1 cells (Fig. 4C). Consistently, miR-17 inhibitor did not increase S1P-induced migration in PTK6-KD ML-1 cells (Fig. 4D). The miR-17 decreases in S1P-induced migration through PTK6 were not cell-type specific because similar results were observed in FTC-133 cells (S5C and D Fig.). Because ERK1/2 lies downstream in the signaling pathway of S1P-induced migration, we sought to determine whether ERK1/2 affected the function of miR-17. In cell migration experiments, overexpression with V12 damaged the function of miR-17 in the migration of ML-1 cells (Fig. 4E). As expected, miR-17 did not affect the migration in ML-1 cells that had been treated with U0126 (Fig. 4E). Similar results were also obtained in FTC-133 cells (S5E and F Fig.). Together, these results demonstrate that miR-17 suppresses S1P-induced follicular thyroid cancer cell migration by PTK6 and ERK1/2.

### PTK6 and miR-17 plays an important role in S1P induced ERK phosphorylation

To define the role of miR-17 in the regulation of S1P induced ERK phosphorylation, ML-1 cells were transfected with pre-miR-17 or pre-control for 45 hours and treated with S1P for 3 hours. Results from Western blot assay showed that ERK1/2 activity was increased by S1P treatment and it was blocked by transfection with pre-miR-17 (S6A Fig.). Similarly, ERK1/2 phosphorylation also abolished with PTK6 knock-down (S6B Fig.). We further examined the effects of PTK6 on miR-17- associated ERK phosphorylation. Western blot assay showed that ERK1/2 activity was increased in the presence of anti-miR-17 but reduced in the presence of siRNA-PTK6#4 (S6C Fig.). Together, these results demonstrate that PTK6 and miR-17 plays an important role in S1P induced ERK phosphorylation.

### S1P inhibits miR-17 expression

**Fig 5:**
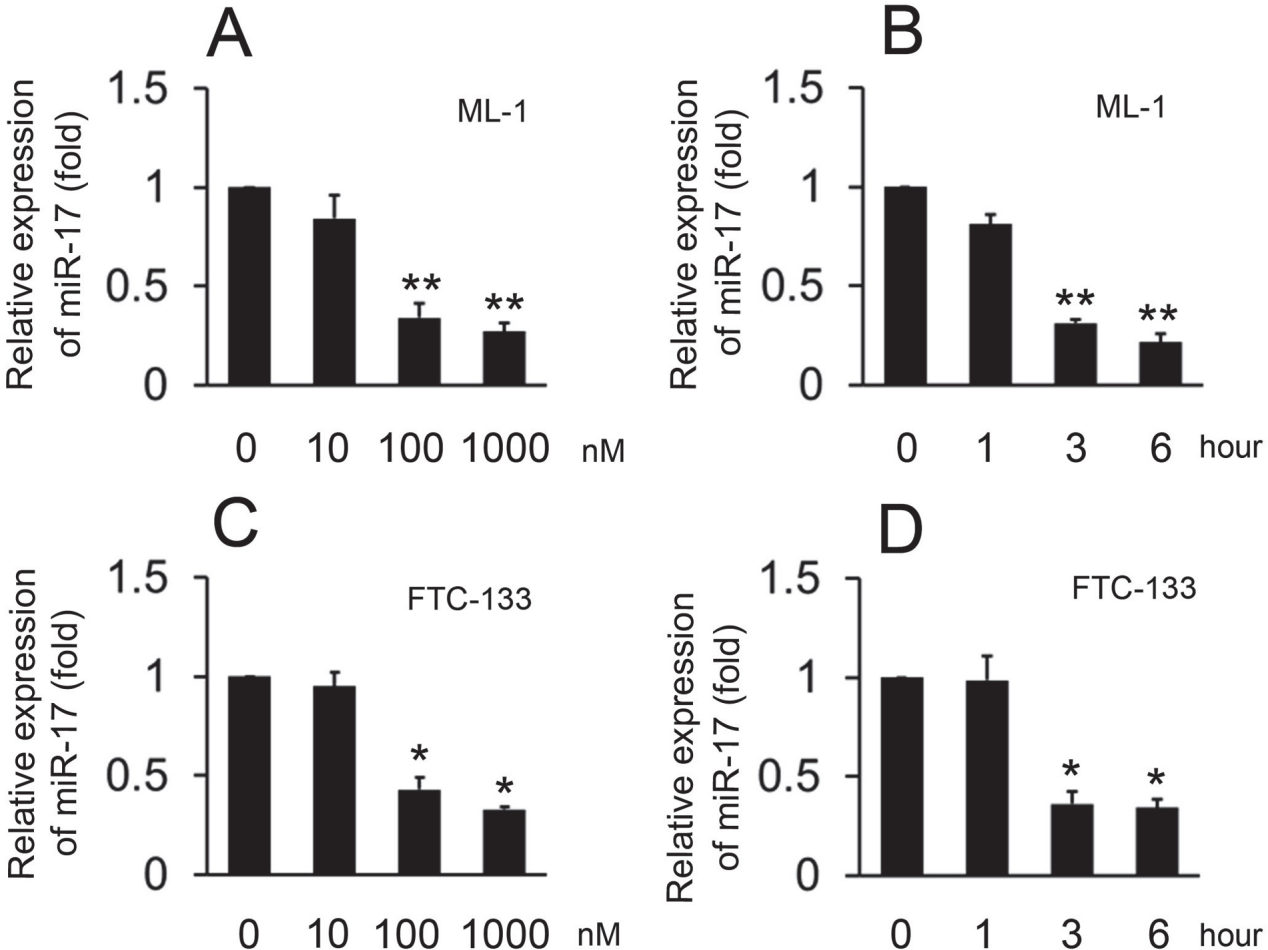
Effect of S1P on the expression of miR-17. Experiments were performed as described in Fig. 1, except miR-17 levels were quantified by real-time RT-PCR. The expression levels were normalized to U6 snRNA. Data represent means±SD, n = 3 (**P < 0.01; *P < 0.05).

To explore the effects of S1P on miR-17 expression, ML-1 cells were treated with various doses of S1P and harvested three hours after treatment. Real-time RT-PCR showed that S1P treatment resulted in decreased miR-17 expression when used at a concentration of 100 nM and resulted in maximal reduction at a concentration of 1000 nM (Fig. 5A). We next treated ML-1 cells with 100 nM S1P and harvested the cells at various timepoints. The reduction of miR-17 expression was detected as early as three hours after treatment with S1P (Fig. 5B). We also repeated the same experiment in FTC-133 cells. The time and dose-related patterns in FTC-133 cells mirrored the data in ML-1 cells (Fig. 5C and D). These data suggest that S1P inhibits miR-17 expression in a time- and dose- dependent manner.

### The expression of PTK6 and miR-17 in papillary thyroid tissues

**Fig 6:**
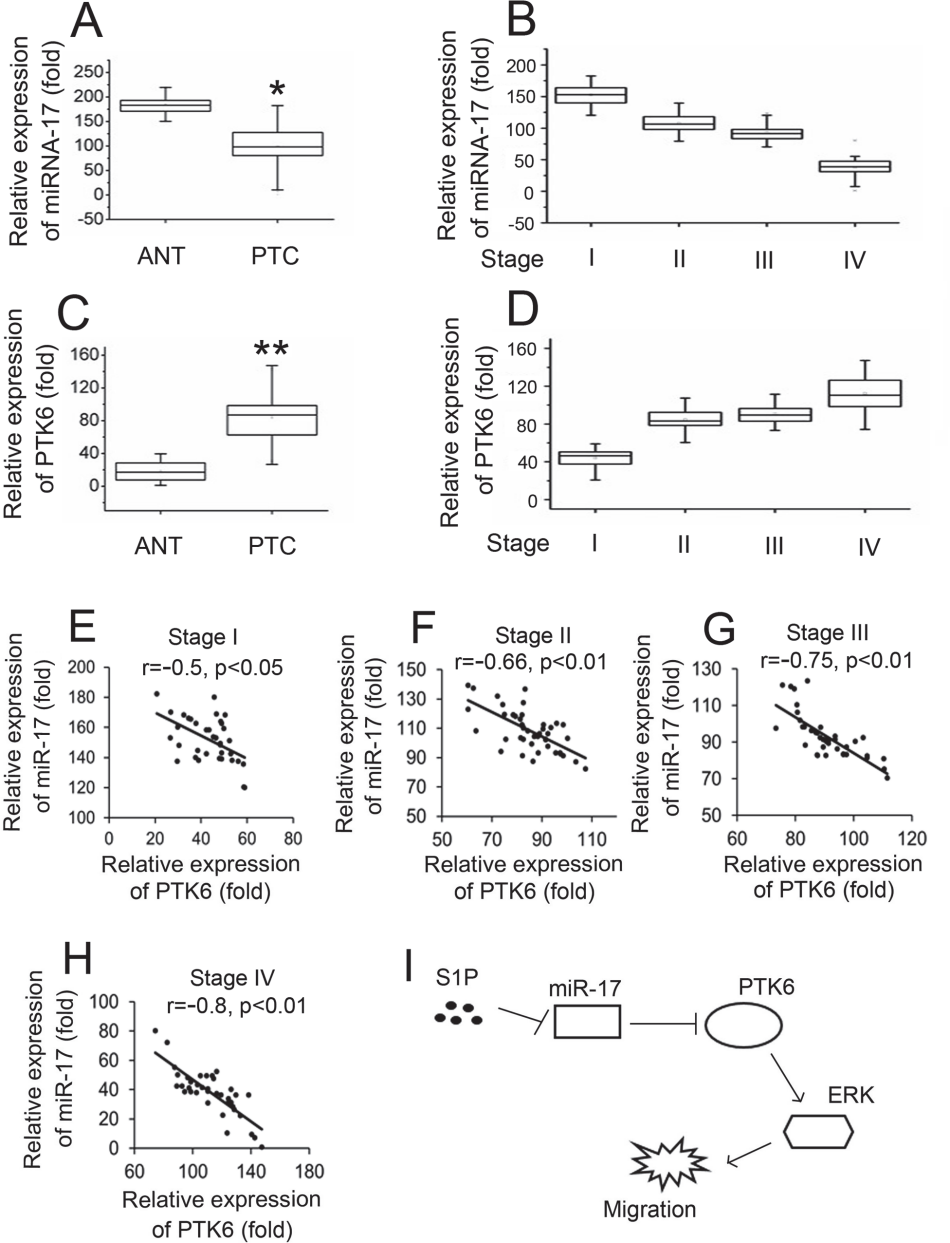
Correlation of PTK6 expression and suppressed miR-17 levels in human follicular thyroid cancer. (A) Real-time RT-PCR assays of miR-17 expression levels in follicular thyroid cancer (FTC) tissues and their corresponding adjacent nontumorous tissues (ANT). The expression of miR-17 was normalized to U6 snRNA. (B) Real-time RT-PCR assays relative miR-17 expression levels in the samples from FTC (stage I, II, III and IV). (C) Real-time RT-PCR assays of PTK-6 expression levels in FTC and their corresponding ANT. The expression of PTK6 was normalized to GAPDH. (D) Real-time RT-PCR assays relative PTK6 expression levels in the samples from FTC (stage I, II, III and IV). (E)-(H) The relative PTK6 mRNA and miR-17 levels in the samples from thyroid cancer tissues (stage I, II, III and IV) were subjected to Pearson’s correlation analysis. (I) A model for regulation of S1P induced migration by miR-17. Boxplots illustrate medians with 25% and 75% and error bars for 5% and 95% percentiles. For A-D, the lowest value was designated as 1. PTK6 or miR-17 data are expressed as fold induction (folds) relative to the lowest value (**P < 0.01; *P < 0.05).

To validate the role of PTK6 and miR-17 in papillary thyroid cancers (PTC), we analyzed PTK6 and miR-17 expression in 162 pairs of clinical PTC and 162 adjacent nontumorous tissues (ANT). As determined by real-time RT-PCR assay, miR-17 was down-regulated in PTC compared to ANT and the expression of miR-17 was progressively decreased from the pTNM stage I to stage IV (Fig. 6A and B). Since PTK6 is the direct target gene of miR-17, we sought to determine whether PTK6 was increased in PTC. Fig. 6C shows that the expression of PTK6 in PTC was significantly higher than in the ANT. And, high levels of PTK6 expression were also associated with pTNM stages (Fig. 6D). Interestingly, elevated PTK6 in the various pTNM stages of PTC was tightly correlated with low levels of miR-17 expression (Fig. 6E, F, G and H). These observations strongly suggest that alterations of PTK6 and miR-17 expression could be involved in thyroid cancer progression.

## Discussion

In this study, we provide a previously undescribed mechanism for S1P regulated thyroid follicular carcinoma cell migration (Fig. 6I). We determined that S1P inhibits miR-17 expression and induces PTK6 expression. Further, studies showed that miR-17 inhibits S1P induced migration of thyroid follicular carcinoma cells by targeting PTK6 and inhibiting ERK1/2 signaling (Fig. 6I).

The expression of PTK6 is very intriguing. PTK6 is uniformly undetectable in benign lesions or in normal mammary tissues, but it is up-regulated in multiple tumor types, including breast, head and neck, ovarian and lung tumours [7, 26–28]. Due to its overexpression in various cancers, more and more studies have tried to uncover the role of PTK6 in the biological behavior of cancers. PTK6 associates with ErbB2 and promotes ErbB2-dependent Ras/MAPK signaling, suggesting PTK6 participates in tumour development [29]. PTK6 can promote STAT3 activation leading to mammary gland tumorigenesis in mouse models [30]. PTK6 also cooperates with HER2 and Src to regulate breast cancer cell survival [31]. Although several functions of PTK6 have been described, a role for PTK6 in papillary thyroid cancer has not been established. In this study we showed that S1P induces PTK6 expression and induces PTK6 activated ERK1/2 signaling, resulting in thyroid follicular carcinoma cell migration. Although, previous studies suggest that PTK6 plays an important role in tumorigenesis and cell proliferation, we did not find evidence to support a role for PTK6 in cancer cell proliferation or apoptosis in papillary thyroid cancer (data not shown). The reasons for this phenomenon need to be explored in future studies.

The function of the miR-17-92 cluster in cancer is controversial and poorly defined. Some studies indicate that the miR-17-92 cluster participates in carcinogenesis as a classical oncogene and is overexpressed in malignant tumors [32–35]. However, one study has shown that the miR-17-92 cluster (especially miR-17, 20a) suppresses oral squamous cell carcinoma migration [36]. Another study found that the miR-17-92 cluster is a tumor suppressor gene, because it regulates the oncogenes E2F1 and MYC to inhibit cell cycle progression [37]. Those data indicate that the miR-17-92 cluster may be a potential tumor suppressor gene. In this work, we showed that miR-17 inhibits S1P-induced migration by directly targeting the 3’-UTR of PTK6. Our results are in line with previously reported findings, although the exact mechanisms exacting remain unclear. MiR-17 belongs to the miR-17–92 cluster, which also includes miR-18a, 19a, 19b-1,20a, 92a-1 [20]. Other miRNAs in the miR-17–92 cluster may also be involved in thyroid follicular carcinoma cell migration. Further studies are needed to verify these putative functions.

In summary, although more studies are needed to understand the delicate regulatory mechanisms of thyroid follicular carcinoma migration, our results provide new insights into the mechanisms responsible for S1P induced migration.

## Supporting Information

S1 Fig S1P affects ERK1/2 activation. (DOC)

S2 Fig Effect of PTK6 and ERK1/2 on S1P-induced migration of FTC-133 cells. (DOC)

S3 Fig Screening for miRNAs that are involved in the S1P-induced signaling pathway and that target the 3’UTR of PTK6. (DOC)

S4 Fig miR-17 down-regulates PTK6 expression in FTC-133 cells. (DOC)

S5 Fig miR-17 inhibits the S1P-induced cell migration through the PTK6-ERK signaling pathway in FTC-133 cells. (DOC)

S6 Fig Effect of miR-17 and PTK6 on S1P-induced ERK phosphorylation. (DOC)

S1 Table Correlation of PTK6 and miR-17 expression with clinicopathologic features in papillary thyroid cancers (PTC). (DOC)

S2 Table Primers Used in Quantitative Real-Time RT-PCR. (DOC)

S3 Table The target sequence of PTK6 and ERK miRNAs. (DOC)
