# Supplementary material for: Sphingosine-1-Phosphate Induces the Migration of Thyroid Follicular Carcinoma Cells through the MicroRNA-17/PTK6/ERK1/2 Pathway
Source: PLoS One. 2015 Mar 6;10(3):e0119148. doi: 10.1371/journal.pone.0119148 (PMC4351951; doi:10.1371/journal.pone.0119148)
Supplement: S1 Fig — (DOC) [file pone.0119148.s001.doc]

**
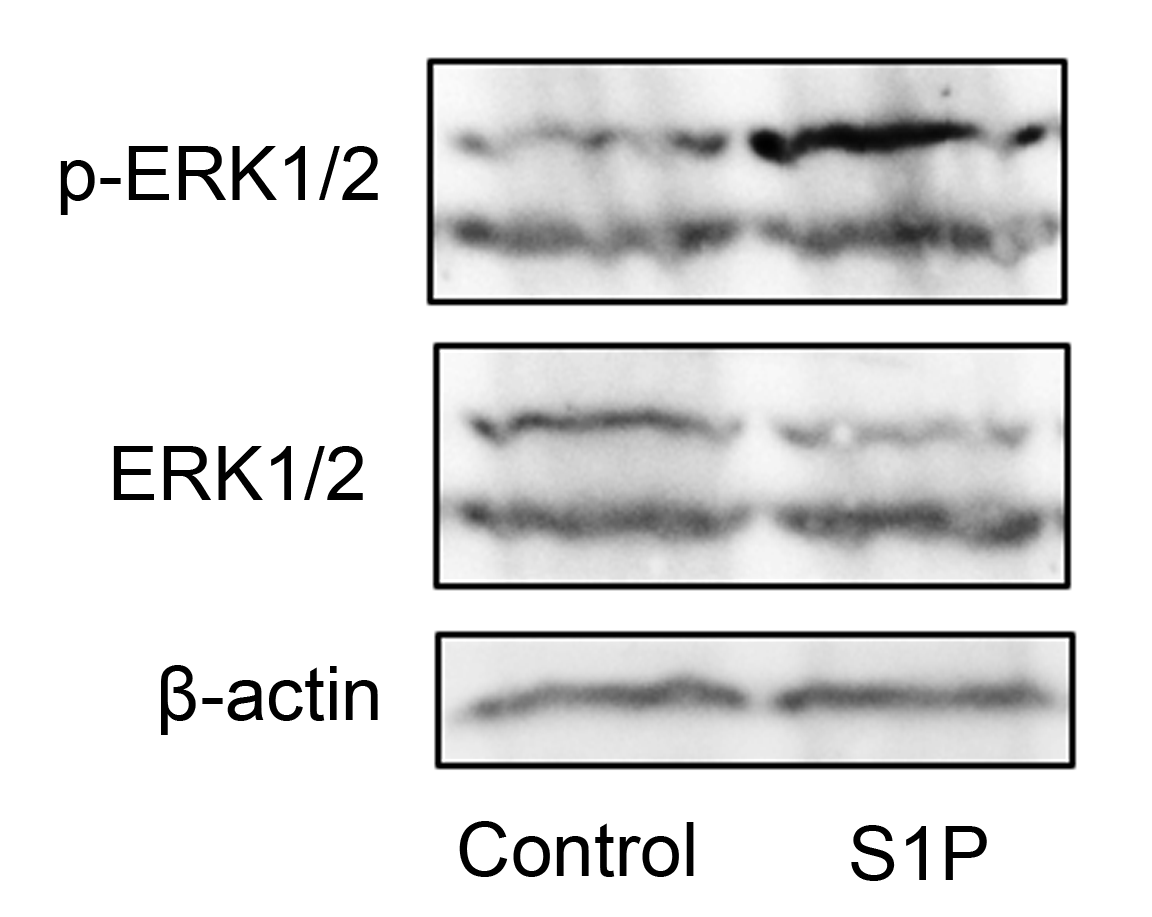
**

**Figure S1. S1P affects ERK1/2 activation.** ML-1 cells were stimulated with 100nM S1P for 3 hours prior to Western blot analysis. Experiments were performed three times with similar results.
