# Supplementary material for: Sphingosine-1-Phosphate Induces the Migration of Thyroid Follicular Carcinoma Cells through the MicroRNA-17/PTK6/ERK1/2 Pathway
Source: PLoS One. 2015 Mar 6;10(3):e0119148. doi: 10.1371/journal.pone.0119148 (PMC4351951; doi:10.1371/journal.pone.0119148)
Supplement: S2 Fig — (DOC) [file pone.0119148.s002.doc]

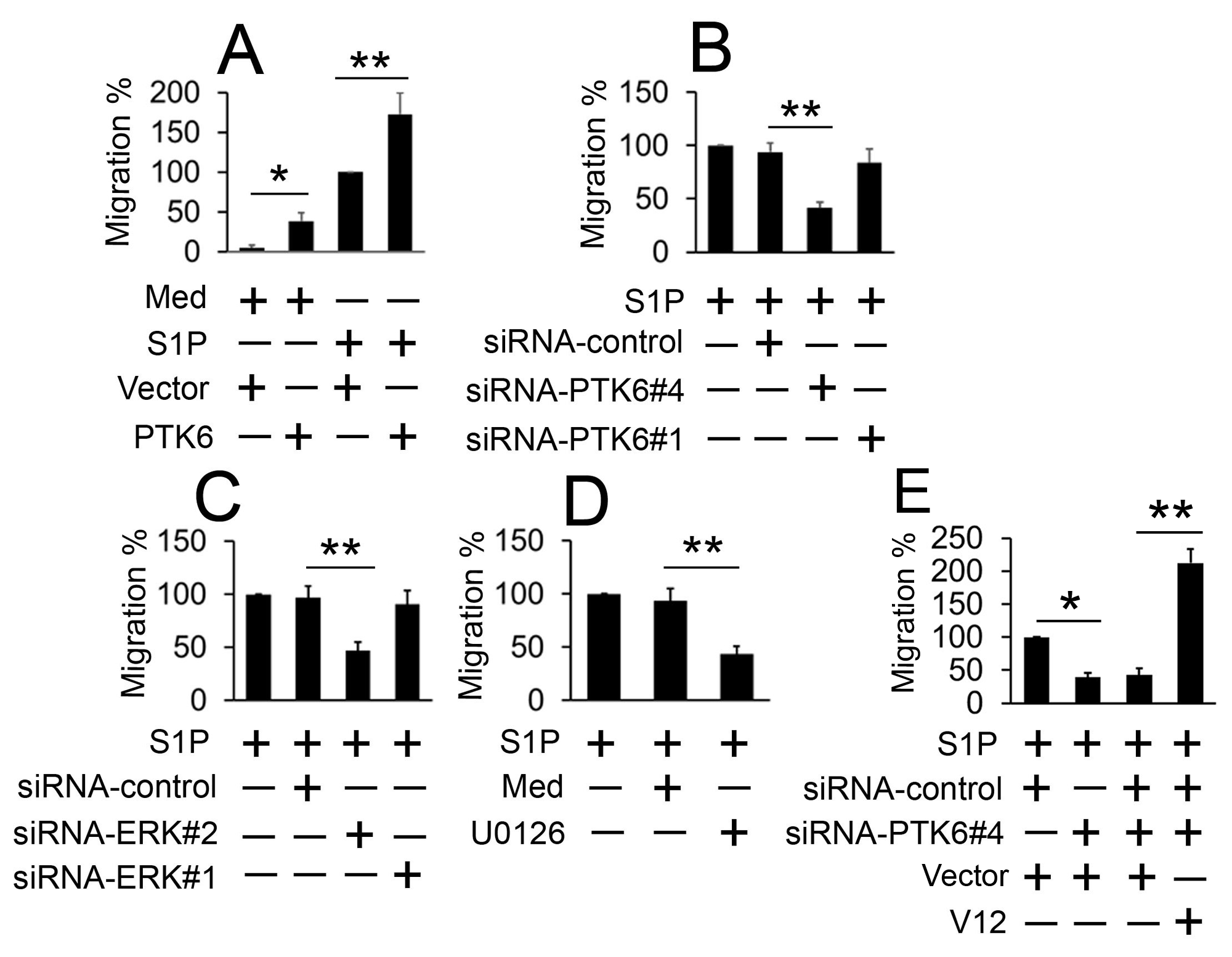


**Figure S2. Effect of PTK6 and ERK1/2 on S1P-induced migration of FTC-133 cells.** (A) and (B) Experiments were performed as described in Fig 2D and Fig 2E except FTC-133 cells were used. (C-E) Experiments were performed as described in Fig 2G, Fig 2H and Fig 2I except FTC-133 cells were used. All experiments were repeated at least three times with similar results. Bar graphs represent means±SD, n=3 (**P < 0.01; *P < 0.05).
