# Supplementary material for: Sphingosine-1-Phosphate Induces the Migration of Thyroid Follicular Carcinoma Cells through the MicroRNA-17/PTK6/ERK1/2 Pathway
Source: PLoS One. 2015 Mar 6;10(3):e0119148. doi: 10.1371/journal.pone.0119148 (PMC4351951; doi:10.1371/journal.pone.0119148)
Supplement: S3 Fig — (DOC) [file pone.0119148.s003.doc]

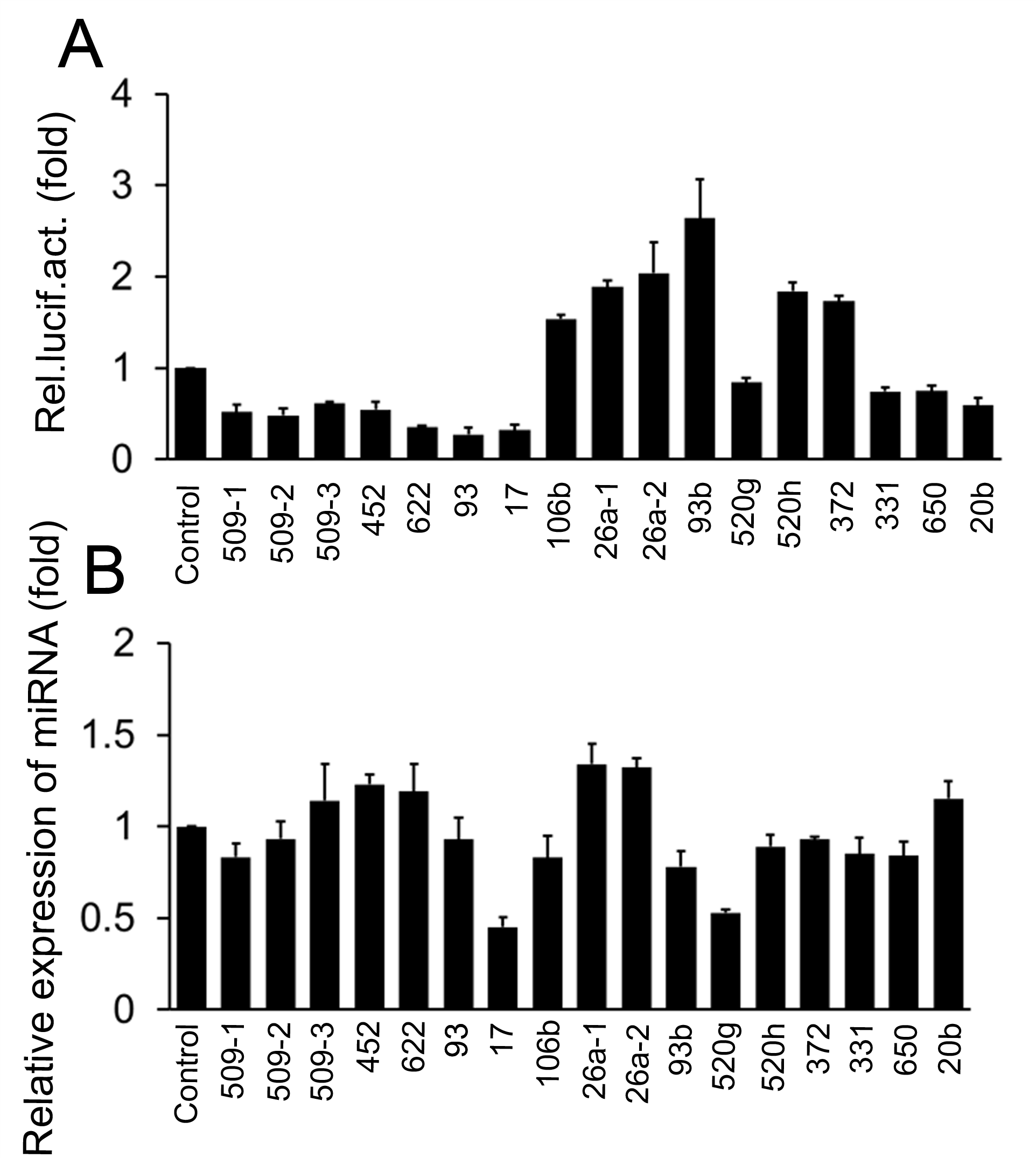


**Figure S3. Screening for miRNAs that are involved in the S1P-induced signaling pathway and that target the 3’UTR of PTK6.** (A) ML-1 cells were transfected with the indicated miRNA and plasmids for 48 hours prior to luciferase assays. (B) ML-1 cells were stimulated with 100nM S1P for 3 hours prior to real-time RT-PCR analysis. Experiments were performed three times with similar results.
