# Supplementary material for: Sphingosine-1-Phosphate Induces the Migration of Thyroid Follicular Carcinoma Cells through the MicroRNA-17/PTK6/ERK1/2 Pathway
Source: PLoS One. 2015 Mar 6;10(3):e0119148. doi: 10.1371/journal.pone.0119148 (PMC4351951; doi:10.1371/journal.pone.0119148)
Supplement: S4 Fig — (DOC) [file pone.0119148.s004.doc]

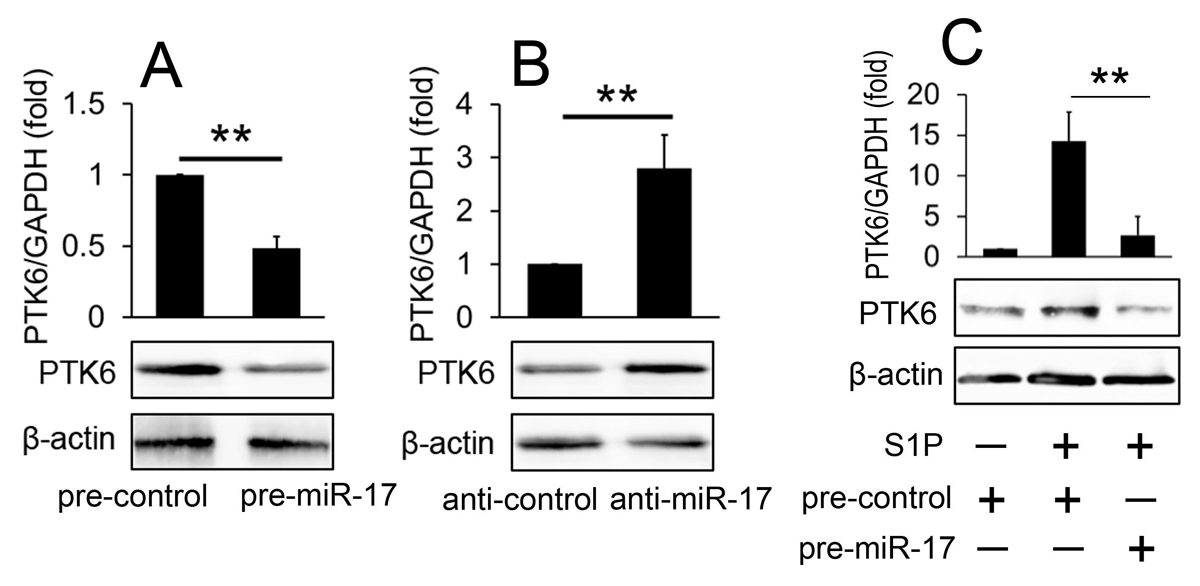


**Figure S4. miR-17 down-regulates PTK6 expression in FTC-133 cells.** (A) FTC-133 cells were transfected with pre-control or pre-miR-17 for 48 hours prior to real-time RT-PCR assays (upper panel) Western blot assays (lower panel). (B) Experiments were performed as in A, except anti-miR-17 was used. (C) FTC-133 cells were transfected with pre-control or pre-miR-17 for 45 hours and were then treated with S1P (100 nM) for 3 hours. PTK6 mRNA levels were quantiﬁed by real-time RT-PCR (upper panel) and protein levels of PTK6 were detected by Western blot (lower panel). All experiments were repeated at least three times with similar results. Bar graphs represent means±SD, n=3 (**P < 0.01; *P < 0.05).
