# Supplementary material for: Sphingosine-1-Phosphate Induces the Migration of Thyroid Follicular Carcinoma Cells through the MicroRNA-17/PTK6/ERK1/2 Pathway
Source: PLoS One. 2015 Mar 6;10(3):e0119148. doi: 10.1371/journal.pone.0119148 (PMC4351951; doi:10.1371/journal.pone.0119148)
Supplement: S5 Fig — (DOC) [file pone.0119148.s005.doc]

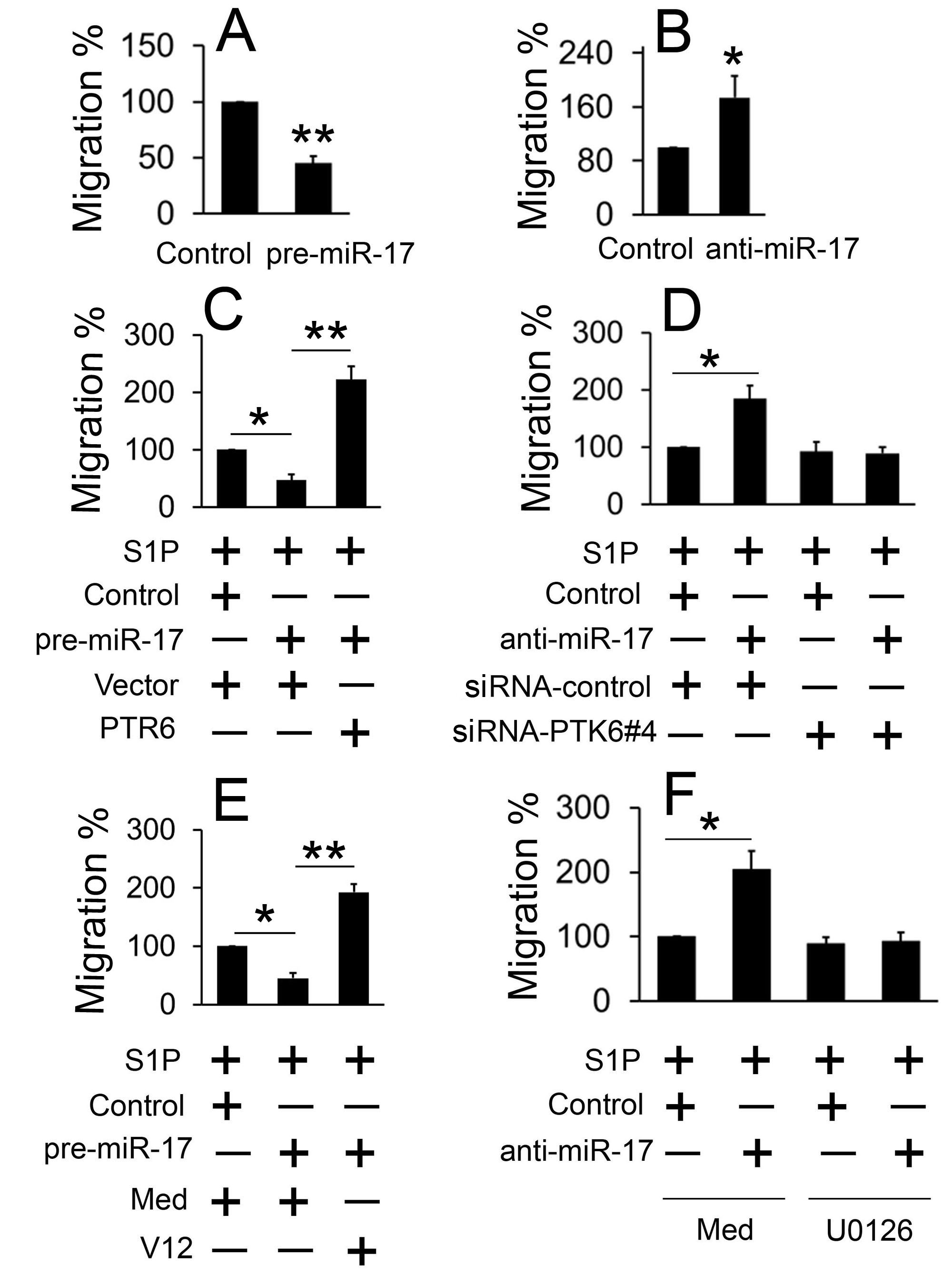


**Figure S5. miR-17 inhibits the S1P-induced cell migration through the PTK6-ERK signaling pathway in FTC-133 cells.** (A-C) Experiments were performed as described in Fig 4A, Fig 4B and Fig 4C except FTC-133 cells were used. (D) FTC-133 cells were transfected with indicated the siRNA and miRNA for 24 hours and reated with S1P (100 nM, 30 min) and allowed to migrate towards serum for 12 hours. (E) and (F) Experiments were performed as described in Fig 4E and Fig 4F except FTC-133 cells were used. All experiments were repeated at least three times with similar results. Bar graphs represent means±SD, n=3 (**P < 0.01; *P < 0.05).
