# Supplementary material for: Sphingosine-1-Phosphate Induces the Migration of Thyroid Follicular Carcinoma Cells through the MicroRNA-17/PTK6/ERK1/2 Pathway
Source: PLoS One. 2015 Mar 6;10(3):e0119148. doi: 10.1371/journal.pone.0119148 (PMC4351951; doi:10.1371/journal.pone.0119148)
Supplement: S6 Fig — (DOC) [file pone.0119148.s006.doc]

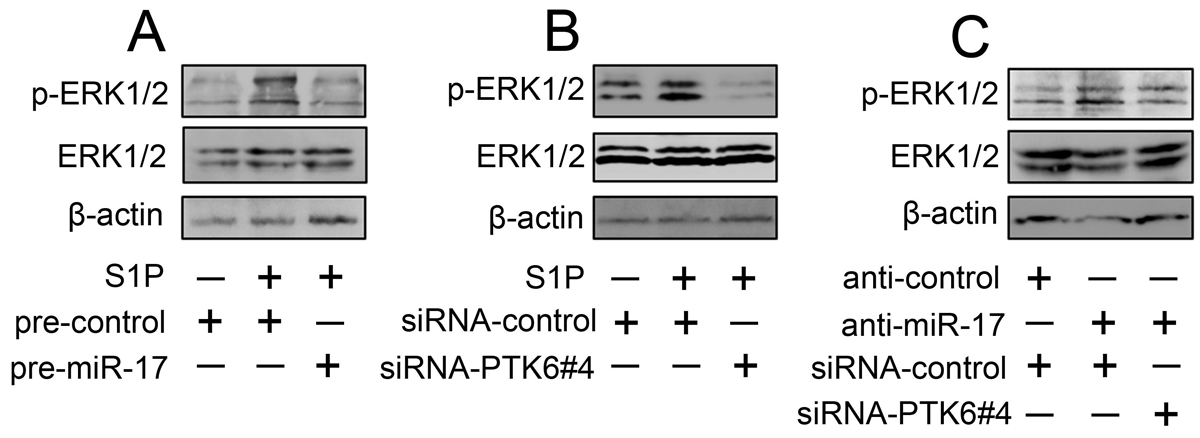


**Figure S6. Effect of miR-17 and PTK6 on S1P-induced ERK phosphorylation.** (A) ML-1 cells were transfected with pre-control or pre-miR-17 for 45 hours, treated with S1P (100 nM) for 3 hours, and subjected to Western blot assays. (B) Experiments were performed as described in A except cells were transfected with indicated siRNA-control or siRNA-PTK6#4. (C) ML-1 cells were transfected with indicated siRNA or miRNA for 48 hours prior to Western blot assays. All experiments were repeated at least three times with similar results.
