# Supplementary material for: Sphingosine-1-Phosphate Induces the Migration of Thyroid Follicular Carcinoma Cells through the MicroRNA-17/PTK6/ERK1/2 Pathway
Source: PLoS One. 2015 Mar 6;10(3):e0119148. doi: 10.1371/journal.pone.0119148 (PMC4351951; doi:10.1371/journal.pone.0119148)
Supplement: S1 Table — (DOC) [file pone.0119148.s007.doc]

Table S1: Correlation of PTK6 and miR-17 expression with clinicopathologic features in papillary thyroid cancers (PTC).

| **Clinicopathologic parameters** | **Case no.** | **PTK6 expression (folds)** | **P value** | **miRNA-17 expression(folds)** | **P value** |
| --- | --- | --- | --- | --- | --- |
| **Age** |  |  |  |  |  |
| ≤50 | 73 | 78.34±10.32 | ns | 107.4±9.32 | ns |
| ＞50 | 89 | 81.23±14.29 |  | 97.3±10.34 |  |
| **Tissue type** |  |  |  |  |  |
| Normal tissue | 162 | 17.76±1.54 | <0.01 | 183.4±17.3 | <0.05 |
| Carcinoma | 162 | 83.08±26.34 |  | 98.4±6.2 |  |
| **Sex** |  |  |  |  |  |
| Male | 70 | 80.23±18.1 | ns | 99.43±10.3 | ns |
| Female | 92 | 76.29±11.32 |  | 95.3±8.32 |  |
| **Tumor size** |  |  |  |  |  |
| ≤5cm | 83 | 32.43±9.32 | <0.05 | 173.2±11.3 | <0.05 |
| ＞5cm | 79 | 98.79±19.3 |  | 62.9±7.3 |  |
| **TNM stage** |  |  |  |  |  |
| Ⅰ | 38 | 43.94±9.88 | <0.05 | 152.5±14.7 | <0.05 |
| Ⅱ | 46 | 84.68±10.99 |  | 107.9±14.4 |  |
| Ⅲ | 40 | 90.6±9.48 |  | 93.3±12.2 |  |
| Ⅳ | 38 | 112.35±17.73 |  | 38.1±15.7 |  |
| **Lymph nodemetastasis** |  |  |  |  |  |
| Negative | 93 | 63.21±7.93 | <0.05 | 164.32±21.96 | <0.05 |
| Positive | 69 | 102.43±10.87 |  | 89.42±11.43 |  |
| **Distant metastasis** |  |  |  |  |  |
| Negative | 124 | 47.43±12.59 | <0.01 | 134.97±25.94 | <0.01 |
| Positive | 38 | 98.53±8.19 |  | 68.17±14.32 |  |
