# Supplementary material for: Sphingosine-1-Phosphate Induces the Migration of Thyroid Follicular Carcinoma Cells through the MicroRNA-17/PTK6/ERK1/2 Pathway
Source: PLoS One. 2015 Mar 6;10(3):e0119148. doi: 10.1371/journal.pone.0119148 (PMC4351951; doi:10.1371/journal.pone.0119148)
Supplement: S2 Table — (DOC) [file pone.0119148.s008.doc]

Table S2: Primers Used in Quantitative Real-Time RT-PCR

| **Target gene** | **5’primer (5’ to 3’)** | **3’primer (5’ to 3’)** |
| --- | --- | --- |
| **GAPDH** | GGAAGGTGAAGGTCGGAGTCAACGG | CTCGCTCCTGGAAGATGGTGATGGG |
| **PTK6** | GGAGTCGCAGAATTACATCCACCG | GCAGGAGAATCCCAAAGGACCAGA |
| **ERK** | TGCCACTCATCATCGGCTCCTCG | GCAAACTCCCGCACTGCCTCGTT |
