# Supplementary material for: Sphingosine-1-Phosphate Induces the Migration of Thyroid Follicular Carcinoma Cells through the MicroRNA-17/PTK6/ERK1/2 Pathway
Source: PLoS One. 2015 Mar 6;10(3):e0119148. doi: 10.1371/journal.pone.0119148 (PMC4351951; doi:10.1371/journal.pone.0119148)
Supplement: S3 Table — (DOC) [file pone.0119148.s009.doc]

Table S3: The target sequence of PTK6 and ERK miRNAs

| **miRNAs** | **target sequence (5’ to 3’)** |
| --- | --- |
| siRNA-PTK6#1 | TACCTCTCCCATGACCACAAT |
| siRNA-PTK6#2 | AGTCGCAGAATTACATCCACC |
| siRNA-PTK6#3 | AGGTGGCCATTAAGGTGATTT |
| siRNA-PTK6#4 | GTGCAGGAAAGGTTCACAAAT |
| siRNA-ERK#1 | CAAAGTTCGAGTAGCTATCAA |
| siRNA-ERK#2 | TGGAATTGGATGACTTGCCTA |
| siRNA-ERK#3 | TATCCATTCAGCTAACGTTCT |
| siRNA-ERK#4 | CCCATATCTGGAGCAGTATTA |
| siRNA-control | GTTCTCCGAACGTGTCACGT |
